# The In Vivo Selection Method in Breast Cancer Metastasis

**DOI:** 10.3390/ijms22041886

**Published:** 2021-02-14

**Authors:** Jun Nakayama, Yuxuan Han, Yuka Kuroiwa, Kazushi Azuma, Yusuke Yamamoto, Kentaro Semba

**Affiliations:** 1 Division of Cellular Signaling, National Cancer Center Research Institute, Tokyo 104-0045, Japan; css124244@akane.waseda.jp (Y.K.); yuyamamo@ncc.go.jp (Y.Y.); 2 Department of Life Science and Medical Bioscience, School of Advanced Science and Engineering, Waseda University, Tokyo 162-8480, Japan; y.han2@kurenai.waseda.jp (Y.H.); a-kazushi@ruri.waseda.jp (K.A.); ksemba@waseda.jp (K.S.); 3 Department of Cell Factory, Translational Research Center, Fukushima Medical University, Fukushima 960-1295, Japan

**Keywords:** metastasis, in vivo selection, highly metastatic cancer cell line, breast cancer, xenograft model

## Abstract

Metastasis is a complex event in cancer progression and causes most deaths from cancer. Repeated transplantation of metastatic cancer cells derived from transplanted murine organs can be used to select the population of highly metastatic cancer cells; this method is called as in vivo selection. The in vivo selection method and highly metastatic cancer cell lines have contributed to reveal the molecular mechanisms of cancer metastasis. Here, we present an overview of the methodology for the in vivo selection method. Recent comparative analysis of the transplantation methods for metastasis have revealed the divergence of metastasis gene signatures. Even cancer cells that metastasize to the same organ show various metastatic cascades and gene expression patterns by changing the transplantation method for the in vivo selection. These findings suggest that the selection of metastasis models for the study of metastasis gene signatures has the potential to influence research results. The study of novel gene signatures that are identified from novel highly metastatic cell lines and patient-derived xenografts (PDXs) will be helpful for understanding the novel mechanisms of metastasis.

## 1. Introduction

Metastasis is a complex event in cancer progression and causes most deaths from cancer. The spreading cancer cells travel from the original primary tumor site to distant organs as a secondary tumor [1]. Stephan Paget proposed the ‘seed and soil’ hypothesis which is important for understanding multiple steps of metastasis [2,3]. The multistep metastatic cascade consists of (1) progressive growth and invasion into local tissue; (2) vascularization/angiogenesis/lymphangiogenesis; (3) premetastatic niche formation; (4) intravasation; (5) survival in the circulation; (6) extravasation; (7) metastatic niche formation; (8) micrometastasis; and (9) metastatic colonization. To clarify the molecular mechanisms of metastasis, it is necessary to discover genetic profiles and examine gene expression (mRNA, miRNA, and lncRNA), proteomics, metabolomics, and cellular events (signal transduction, cell-cell interactions) at each process of metastatic cascades [4,5,6].

In the methodological history of metastasis research, Makoto Takahashi developed the first murine model for metastasis, in which cancer cells were injected into the tail vein of mice as a lung metastasis model [7]. The injection of cancer cells into metastatic target organs mimics metastatic events and the microenvironment in the mouse body. Isaiah J. Fidler developed the mouse melanoma cell lines, B16 and its B16-F10 (which have a high metastatic activity to the lung), using tail vein injection into an allograft model and tissue culture techniques [8]. Repeated injection of metastatic cancer cells derived from transplanted murine organs selects the population of highly metastatic cancer cells; this method is called in vivo selection. The in vivo selection method and established highly metastatic cancer cell lines have contributed to our understanding of the molecular mechanisms of cancer metastasis.

The establishment of immunodeficient mice such as nude, SCID, NOD, NOD-SCID, NOG, and NSG mice contributed to xenograft studies for metastasis using human cancer cell lines. I. D. J. Bross et al. showed that metastatic spreading occurred in immunodeficient mice by using xenografts of human myeloma and leukemia [9]. Although the greatest problem associated with xenografts is immune rejection, the loss of innate immunity and adaptive immunity in mice by genetic manipulation has enabled the transplantation of cells derived from other species, such as humans [10]. Chloe C. Milsom et al. showed that severely immunodeficient mice were prone to metastases [11]. It has become easier to carry out metastatic studies in graft models. However, problems associated with the graft model include the loss of metastatic interactions with the immune system and the uniqueness of the grafted tumor tissue. Patient-derived xenografts (PDXs) were expanded with the development of immunodeficient mice despite the poor engraft rate, and this model better maintains the uniqueness of the grafted tumor tissue than a xenograft model using a cancer cell line [12,13].

Genetically engineered mouse models (GEMMs) mimic spontaneous metastasis formation from primary tumor [14,15]. In addition, the CRISPR/Cas9 system boosted metastasis research using a GEMM [16]. However, previous research has reported that it is difficult to mimic the clinical metastatic pattern and the clinical site of relapse using a GEMM [12]. On the other hand, graft models can mimic various metastatic patterns and steps of metastasis cascade using an appropriate injection method. Highly metastatic cell lines and graft models are enabled to reveal the mechanism of metastasis. Graft models and highly metastatic cancer cell lines are still commonly used in current studies, and their contribution to metastasis research is significant [17,18,19,20,21]. Here, we present an overview of metastasis biology using highly metastatic cell lines established by the in vivo selection method. This review focuses on the characteristics of highly metastatic breast cancer cell lines with the viewing of methodology for establishment.

## 2. Differences in Transplantation Methods for the In Vivo Selection

### 2.1. Differences in Highly Metastatic Breast Cancer Cell Lines

In this section, we discuss that the methodological differences in breast cancer metastasis are important information to select relevant metastasis model. A spontaneous breast cancer metastasis model was generated through orthotopic transplantation into mammary fat pads. This orthotopic model has helped to reveal molecular mechanisms of cancer metastasis and progression [22,23,24]. Andy J Minn et al. showed that the knockdown of interleukin 13 receptor subunit alpha2 (*IL13RA2*), secreted protein acidic and cysteine-rich (*SPARC*) and vascular cell adhesion molecule 1 (*VCAM1*) in MDA-MB-231-LM2 cells (derived from MDA-MB-231-4175 cells) decreased lung metastatic ability after intravenous injection, but these effects did not involve orthotopic tumor growth. On the other hand, knockdown of inhibitor of DNA binding 1 (*ID1*) caused a significant reduction in both [25]. Their findings suggest that there are two types of metastasis-promoting genes; one promotes cancer progression in only a metastatic site, and the other leads to progression in both orthotopic and metastatic sites.

Recent studies have reported a relationship between the transplantation method for in vivo selection and metastasis gene signatures. Jun Nakayama et al. showed the differences between orthotopic transplantation and intravenous injection via the establishment of a lung metastatic breast cancer cell line. They established lung metastatic MDA-MB-231 cells by two methods—orthotopic transplantation and intravenous injection. The gene signatures for the established metastatic cells were remarkably different. Their model showed that chemotaxis and cell adhesion genes were enriched in the orthotopic lung metastasis model [26]. In addition, Nir Pillar et al. showed that microRNA expression profiles are different between orthotopic transplantation and intravenous injection [27]. Christina Ross et al. performed a comparative analysis of orthotopic transplantation and intravenous injection, allograft models, and GEMMs. Interestingly, their study confirmed not only the divergence in gene signatures between routes of the transplantation model, but also that the extent of this variation is unique to each cell line. Gene signatures in the orthotopic transplantation are enriched in T-cell activation and immune-related pathways. In addition, allograft models showed more mesenchymal-like expression than GEMMs [28].

These studies suggest that the selection of breast cancer metastasis models for the study of metastasis gene signatures has the potential to influence research results. Many gene signatures in orthotopic breast cancer metastasis have not yet been studied, and we believe that these studies will contribute to the elucidation of novel mechanisms of breast cancer metastasis and the development of a cure for breast cancer metastases. Although recent studies show a novel potential of cancer cell line studies [29], generally most findings from cancer cell line models are difficult to translate into clinical practice [30]. Cancer cell lines have the limitation in preclinical research, since cancer cell lines have acquired the genetic mutations which enable them to proliferate in a plastic dish [31,32]. On the other hand, PDX models have shown to be clinically relevant in comparison to breast cancer cell lines [33].

### 2.2. Breast Cancer Metastasis in PDX Models

PDX models are superior to cancer cell line xenograft models and GEMMs, since PDX models reflect the diversity and heterogeneity of human tumors [34,35]. In breast cancer research, PDX models of each breast cancer subtype are developed for preclinical studies [33]. Recent studies reported that 3887-LM, which is a highly lung metastatic basal-like breast cancer PDX model, is developed by serial transplantation as an in vivo selection method [36] (Table 1). Adrián González-González et al. reported that SMAD2/3/4 and mTORC2 signaling mediate lung metastasis in a 3887-LM PDX model [37]. Marta Paez-Ribes et al. established HCI-002 LM2-1 (highly lung metastatic) triple-negative breast cancer (TNBC) PDXs model by serial orthotopic transplantation [38]. Masanori Oshi et al. established novel brain metastasis models in TNBC PDX by orthotopic transplantation and intracranial injection method [39]. Interestingly, Diane Lefley et al. showed the novel bone metastasis model using a PDX model with bone tissue derived from humans [40]. Their metastasis model can mimic a human bone microenvironment in immunodeficient mice, and cancer cells can metastasize to human metastatic organs. Since PDX models of tumor cells and metastatic organs have established, it may be possible to observe human cancer metastasis in mice.

However, PDX models also have technical problems of the establishment process. Major problems are the high cost for maintenance and a low ratio of engraftment of tumor tissues derived from patient. Additionally, the engraftment sites of the xenograft also affect the success rate of establishment of PDXs [41]. Since the transplantation methods have effects on the metastasis gene signatures and the characters of cancer cell, study is needed for elucidating the differences in the PDX character by each engraftment site.

## 3. Highly Metastatic Breast Cancer Cell Lines

### 3.1. Introduction of Highly Metastatic Breast Cancer Cell Lines

**Figure 1:**
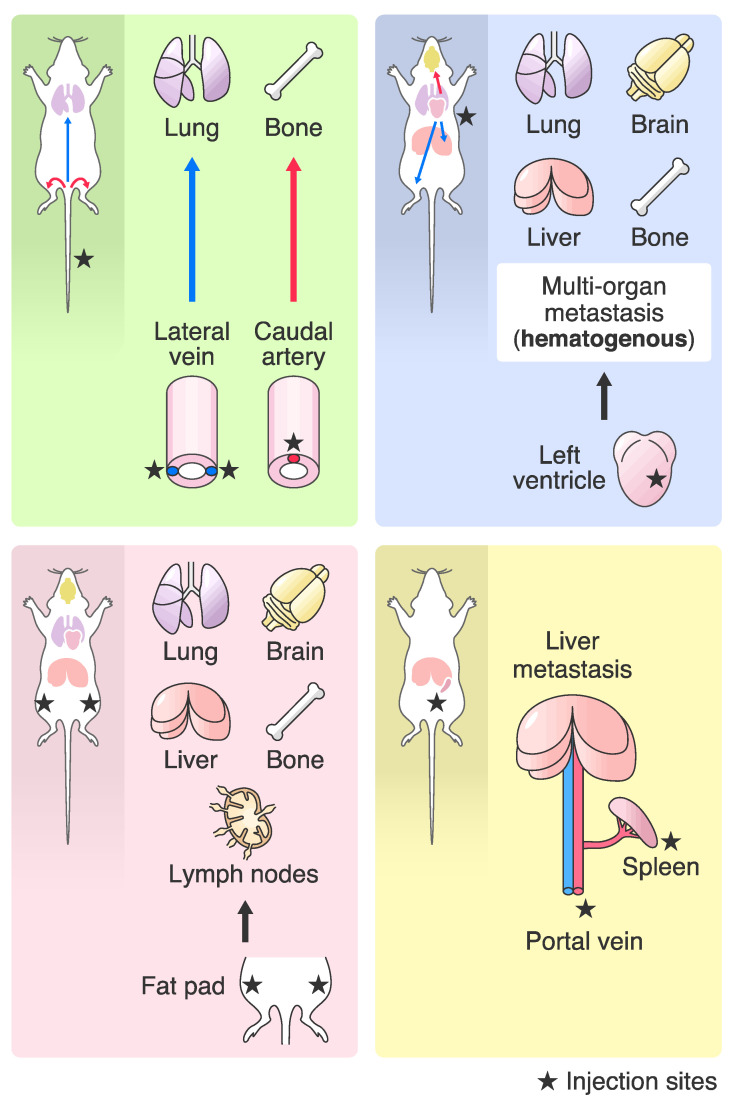
Transplantation methods in tropic organs. Tail vein injection (**upper left**), intracaudal artery injection (**upper left**), intracarotid injection and intracardiac injection (**upper right**) are categorized as intravascular transplantation. These methods can deliver cancer cells to distant organs via the blood circulation and can be used to generate a hematogenous metastasis model (study of micrometastasis formation and metastatic colonization in distant organs, extravasation process and metastatic niche formation). The orthotopic transplantation method can mimic tumor progression from the primary tumor to distant organs and be used as a hematogenous or lymphogenous metastasis model (**lower left**) (study of micrometastasis formation and metastatic colonization in distant organs, extravasation process, metastatic niche formation, survival in the circulation, intravasation, preparation of the pre-metastatic niche, vascularization/angiogenesis/lymphangiogenesis and progressive growth and invasion into local tissue). The spleen transplantation method can be used to generate a liver metastasis model via hematogenous metastasis (**lower right**) (study of micrometastasis formation and metastatic colonization in distant organs, extravasation process, metastatic niche formation, survival in the circulation, intravasation).

In a clinical study, microarray analysis revealed the heterogeneity and diversity of gene expression in breast cancer patients, and gene signatures were correlated with a poor prognosis [42,43,44]. On the other hand, in an experimental metastasis study, Yibin Kang et al. performed an expression analysis of highly metastatic human breast cancer cell lines established by in vivo selection and revealed a metastatic molecular signature [45]. In addition, Andy J Minn et al. found that gene signatures from highly metastatic human cell lines were correlated with poor progression in breast cancer patients [25]. Breast cancer metastasizes to distant multiorgan sites, such as the lung, bone, brain, and liver, via hematogenous or lymphatic metastases. An analysis of highly metastatic cancer cell lines that have metastatic organ tropism has also demonstrated metastasis-promoting genes that contribute to organ tropism [1,46,47]. Since this discovery, many metastasis-promoting genes, suppressor genes and molecular mechanisms of metastasis have been identified from the analysis of gene signatures of highly metastatic cell lines [48,49]. Although the highly metastatic cell lines that are frequently used are limited, many metastatic cell lines have been established by various transplantation methods (Figure 1 and Table 2) [50,51,52,53,54,55,56,57]. In addition, to promote the exchange of information on these valuable resources, the brain metastasis cell lines panel (BrMPanel) [58] and the metastasis map (MetMap) of human cancer cell lines [59] has been published as a useful public dataset. Cataloging the metastatic tropism and activity of cancer cell lines has reached a major turning point by these datasets [58,59,60]. Here, we describe a study of metastasis biology with gene expression profiling using highly metastatic breast cancer cell lines established by in vivo selection.

### 3.2. Lung Metastasis

The intravenous injection (tail vein injection) method is frequently used to generate lung metastasis models [7]. Since the primary tumor forms long after orthotopic transplantation into mammary fat pads, the intravenous model is convenient for the study of lung metastasis. Andy J. Minn et al. established MDA-MB-231-LM2 cells (lung metastatic cells) [25], as well as CN34-LM1 [66] and MDA-MB-231-HM cells [68] by intravenous transplantation. These three cell lines are the most frequently examined cell lines in breast cancer lung metastasis studies. However, there are other highly lung metastatic cell lines, such as MDA-MB-231-LM1-2-1 (tail vein transplant), and MDA-MB-231-LM05 (orthotopic transplant) [26]. Moreover, the C3L5 cell line (subcutaneous transplant) is a highly lung metastatic cell line derived from a spontaneous mammary tumors [65].

In previous studies using MDA-MB-231-LM2 cells, epiregulin (*EREG*), *C-X-C* motif chemokine ligand 1 (*CXCL1*)*,* matrix metalloproteinase 1 (*MMP1*), *MMP2*, *SPARC, VCAM1,* and cyclooxygenase 2 (*COX2*) were elucidated as genes that mediate lung metastasis [25]. Furthermore, the expression changes in genes related to metastasis initiation (cathepsin C: *CTSC,* endoglin: *ENG,* bone morphogenetic protein 2: *BMP2*) and metastasis suppression (cystatin 1: *CST1*, *CST2*, sodium channel non-voltage-gated 1: *SCNN1A*, *BMP4*) have also been confirmed [75]. Tenascin C (*TNC*), acetylgalactosaminyltransferase 14 (*GALNT14*) and *CD70* are known as genes related to lung-specific metastasis. *TNC* promotes NOTCH and WNT signaling and cancer cell survival in the early stage of lung metastasis and then promotes lung-specific metastasis [76]. The expression of *GALNT14* is induced by the KRAS-PI3K pathway, and increasing of SRY-box 4 (*SOX4*) expression by O-GalNAcylation of the BMP receptor. It has been reported that the self-renewal ability of MDA-MB-231-LM2 and CN34-LM1 cells is enhanced by inhibiting the suppressive effect of lung-derived BMPs and, therefore, promotes cell division [77]. In addition, MDA-MB-231-LM2 and CN34-LM2 cells highly express *CD70*, which is also highly expressed in clinical samples with lung metastases. Although the detailed function of *CD70* in lung metastasis has not yet been reported, *CD70* is one of lung metastasis gene signatures [78]. Moreover, multiple genes are sometimes required to promote lung metastasis, rather than independent genes.

Furthermore, decreases in miR-335, miR-206, and miR-126 expression [66] and increases in circIRAK3 expression [79] were observed as changes in non-coding RNA expression levels. miR-335 regulates the expression of several genes such as *TNC* and *SOX4*, as described above [66]. However, most of the metastasis-related genes and miRNAs that have been discovered are derived from TNBC. There is still a lack of studies on other subtypes of breast cancer.

### 3.3. Bone Metastasis

Bone metastasis is not lethal; however, it remains incurable by any available clinical treatments and causes a series of skeletal-related events [80], induces severe pain in patients, and directly affects quality of life (QOL) [81]. One concept is that breast cancer cells invading the bone are usually accompanied by interactions with osteoblasts and osteoclasts [82]. Breast cancer cells respond to parathyroid hormone-associated protein (PTHrP), converting transforming growth factor beta (TGF-β), cytokines, chemokines, and other growth factors in the bone microenvironment, and mediate the tumor invasion and colonization. Several in vivo injection methods, including orthotropic transplantation, tail vein injection, intracardiac injection, and hind limb injection, can be used to study bone metastasis. Orthotropic models are usually used to study spontaneous metastasis [83], in which metastases have more difficulty forming in the bone than in soft tissue. As the tail vein injection results in a similar phenotype, it is more often used to study lung metastasis than bone metastasis. Intracardiac injection into the left ventricle is the most common technique employed to study organ-tropic metastasis and to establish bone metastatic cell lines [84]. A recent study reported the application of intracaudal arterial injection to establish bone metastatic cell lines [51]. After all, bone metastasis models are more difficult to generate than lung and liver models.

Yibin Kang et al. first applied MDA-MB-231 cells to establish bone metastasis sublines by intracardiac injection. Interleukin 11 (*IL11*) and connective tissue growth factor (*CTGF*) activate osteolytic factors, and their expression is further increased by TGF-β in MDA-MB-231 bone metastasis cells (MDA-MB-231-BM cells; intracardiac injection) [45]. Increased C-X-C motif receptor 4 (*CXCR4*) expression is present in breast cancer cells that have metastasized to the bone through hypoxia inducible factor 1 alpha subunit (*HIF1A*) and TGF-β signaling [85]. A model in which MDA-MB-231 cells were injected into the hind limb musculature proved that *MMP1* released by metastatic cells activates osteoclast precursor cells and improves osteoclast resorption [86]. MDA-MB-231-BM cells release dickkopf 1 (*DKK1*) to increase the serum level and seed bone metastasis through Wnt signaling [87]. E-selectin promotes the mesenchymal-to-epithelial transition process and bone-specific metastasis [74]. Bone metastatic MDA-MB-231 cells (mtMDA) established by intracardiac injection promoted the secretion of S100 calcium binding protein A4 (*S100A4*), which participates directly in the formation of osteolytic lesions of bone metastases [73].

Moreover, the highly metastatic 4T1E/M3 murine breast cancer cell line (intravenous injection) established by intracardiac injection showed upregulated intracellular adhesion molecule 1 (*ICAM1*) and its function in tumor colonization [63]. The 4T1.3 cell lines (highly bone metastatic subline) were established by orthotopic transplantation, which expresses C-C motif ligand 4 (*CCL4*) for interaction with fibroblasts expressing C-C motif receptor 5 (*CCR5*) in the bone microenvironment [62]. Most of bone metastatic cell lines also are established from TNBC. Yuxuan Han et al. established luminal bone metastatic cell lines (MCF7-BM02) by intracaudal arterial injection [67]. These cell lines will be a reasonable model for bone metastasis in luminal breast cancer.

### 3.4. Brain Metastasis

Breast cancer has the second highest incidence of brain metastasis following lung cancer [88]. Patients with brain metastasis have several neurological symptoms, such as headache, cognitive impairment, and convulsion, which worsen the patient’s QOL [89]. However, only a few strategies can be used to specifically treat brain metastasis, and in most cases, therapy for brain metastasis is palliative care [90]. To elucidate the mechanism of brain metastasis for the development of effective treatments, highly metastatic brain cell lines have been established, and their gene expression profile has been determined. Graft models of brain metastasis are generated mainly by four methods—intracardiac injection, intracarotid injection, intracranial injection, and intravenous injection [53,91,92,93,94]. However, most highly brain metastatic cell lines have been established by the intracardiac injection method.

Most studies on brain metastasis in breast cancer have been conducted using the brain metastatic derivatives of TNBC cell lines. The highly brain metastatic cell lines MDA-MB-231-BrM2 and CN34-BrM2 cells were established via the intracardiac injection method [91]. Gene expression analysis of these cells revealed that *COX2*, heparin binding EGF like growth factor (*HBEGF*), and ST6 N-acetylgalactosaminide alpha-2,6-sialyltransferase 5 (*ST6GALNAC5*) were involved in brain metastasis of breast cancer by mediating cancer cell passage through the blood-brain barrier (BBB) [91]. Cathepsin S (*CTSS*), some types of serpins (inhibitors of plasminogen activators), cell adhesion molecule L1 (*L1CAM*), glutamate receptor ionotropic N-methyl D-asparate 2B (*GRIN2B*), protocadherin 7 (*PCDH7*) and connexin 43 (*CX43*) were also reported to promote breast to brain metastasis, as revealed by the expression analysis of brain metastatic cells derived from MDA-MB-231 cells [18,95,96,97,98]. In addition, highly brain metastatic MDA-MB-231-BMD2a and BMD2b cell lines, were established by repetitive intracardiac injection of MDA-MB-231-luc-D3H2LN cells [99].

TNBCs, as well as HER2-positive breast cancers, are prone to metastasize to the brain [100]. The MDA-MB-231-BR-HER2 and MCF7-HER2-BR3 cell lines are used as a model of brain metastasis [19,101,102,103,104]. Additionally, the BT474.Br cells (derived from BT474-m1, intracarotid transplantation) [105,106,107], SKBRM cells [108,109], JIMT-1-BR3, SUM-190-BR3 cells (intracardiac injection) [110,111,112], MDA-MB-361-BR2/BR3 cells (intracardiac injection) [113] and HCC1954-BrM1 cells [93] are often used for the study of brain metastasis in HER2-positive breast cancer.

Regarding murine TNBC cell lines, the brain-metastatic variants of 4T1 cells are often used. The 4TBM cells (the brain-metastatic derivative of heart-metastasized 4T1 cells) were established by the orthotopic injection method [114]. For the murine HER2-positive breast cancer cell line, the brain metastatic cells expressing avian erythroblastosis oncogene B2 (*ErbB2*) (ErbB2-BrM2) were established from MMTV-driven NeuNT transgenic murine mammary tumor cells by repetitive in vivo selection using the intracardiac injection method [93,96,115]. Various brain tropic breast cancer cell lines have been established. Several genes associated with brain metastasis have been identified by gene expression profiling of those cell lines.

### 3.5. Liver Metastasis

Liver metastases are observed in approximately 50% of metastatic breast cancer patients [116]. Patients with breast-to-liver metastasis have unfavorable prognoses and efficacious treatments for liver metastasis are required [117,118]. For liver metastasis in breast cancer, few highly metastatic cell lines have been established so far. MDA-MB-231HMLNm5 cells are highly metastatic cells with a metastatic potential not only to the liver but also to the lung, axillary lymph nodes, spleen, and paraspinal tissue [69,70,71,75,119,120].

Murine mammary liver metastatic cell lines are often used. In many cases, 4T1-derived liver metastatic cells have been used to study liver metastasis in breast cancer. Liver metastatic 4T1 (2776, 2792, and 2869 subclones) cell lines were established by spleen transplantation as the in vivo selection method [61]. Comparative analysis of gene expression profiles between lower liver metastatic cells and highly liver metastatic cells revealed that claudin 2 (*CLDN2*) was upregulated in the aggressive populations and that it promoted liver metastasis by increasing cancer cell adhesion to extracellular matrix components via integrin complexes [61]. In addition, Nuray Erin et al. established liver metastatic 4T1 cells (4TLM cells) from heart-metastasized 4T1 cells (4THM cells) by orthotopic transplantation and compared the gene expression profile of 4TLM cells with that of their parental cells (4T1 cells) [64]. In 4TLM cells, the gene signatures involved in cell adhesion and cell junctions were downregulated [64]. The *CD34* gene is highly expressed in 4TLM primary tumors, reflecting their metastatic potential [114]. Highly liver metastatic 4T1 cell lines have contributed to the research on hepatic metastasis in breast cancer. However, regarding human cell lines, only a few metastatic cell lines have been established in the field of hepatic metastasis. The establishment of the novel liver metastatic human breast cancer cell lines will be helpful for developing an effective treatment for hepatic metastasis.

### 3.6. Lymph Node Metastasis

Metastasis to the sentinel lymph node often occurs in breast cancer. The lymphatic pathways in the human breast include the axilla, internal breast, and supraclavicular lymph node, and are related to distant organ metastasis. Since lymph nodes can also access the blood circulation via lymphatic vessels, metastasis to lymph nodes has the potential to induce subsequent systemic metastasis [72,121]. Many types of studies on lymph node metastasis of breast cancer are based on clinical specimens.

Most of highly lymph node metastatic breast cancer cell lines have been established by orthotopic transplantation. This is because the intravascular injection method cannot imitate the processes of lymphogenous metastasis in mice. MDA-MB-468LN [72], MDA-MB-231-luc-D3H2LN [50], MDA-MB-231H-RFP [122], and MDA-MB-231-HM.LNm5 [69] cell lines were established as lymph node metastatic cell lines from the TNBC cell lines. All these cell lines metastasize to axillary lymph nodes after orthotopic transplantation. Gene expression analysis showed that the mRNA levels of metallothionein 1A (*MT1A*)*, MT1E, MT1M,* and *MT2A* are high and that those of collagen type VI alpha 1 (*COL6A1*)*, COL6A2,* and *COL18A1* are suppressed in MDA-MB-231-HMLNm5 cells [75].

## 4. Differences in Metastasis Gene Signatures According to the Molecular Subtypes of Breast Cancer

The intrinsic subtypes of breast cancer are classified based on the gene expression profiles of hormone receptors (HRs) (estrogen receptor, ER; progesterone receptor, PR) and human epidermal growth factor receptor 2 (HER2). There are four subtypes—luminal A, luminal B, HER2/ERBB2-overexpressing, and TNBC [123]. Subtype classification is very important in determining the treatment strategy for breast cancer.

**Figure 2:**
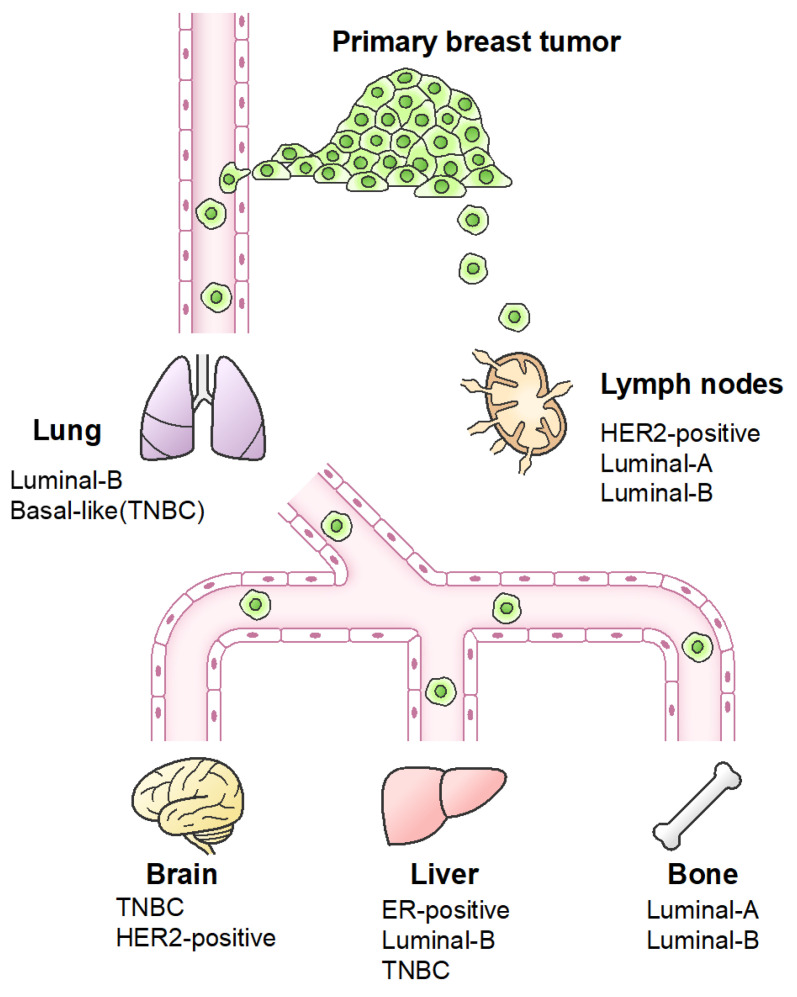
Breast cancer subtypes and tropism of distant metastases. The breast cancer subtypes are classified based on estrogen receptor (ER), human epidermal growth factor receptor 2 (HER2), and progesterone receptor (PR). There are differences of metastatic organ tropism between breast cancer subtypes.

The site of metastasis tends to differ depending on each subtype’s metastatic tropism (Figure 2) [124]. Breast cancer patients of the luminal type (HR+/HER−) show more than twice as much bone metastasis than those of the other subtypes [125,126]. In contrast, TNBC tumors have a high overall rate of brain, liver, and lung metastases [118,126,127,128,129]. Each subtype of breast cancer exhibits different metastatic behaviors to the sites of distant metastasis [129]. Unique gene signatures may be involved in the organ-specific metastasis according to the breast cancer subtype [130].

Therefore, metastasis gene signatures identified from TNBC may not be able to regulate the metastatic process of other subtypes. This raises the crucial problem of treating metastasis, in that the molecular mechanism of metastasis differs not only by the organ site but also by the cancer subtype. For example, the metastatic gene *COX2* is undetectable in MCF7 cells but is overexpressed in MDA-MB-231 cells [131]. Runt-related transcription factor 2 (*RUNX2*) knockdown in MDA-MB-231 cells downregulates the expression of the bone-related genes to induce bone metastasis. However, *RUNX2* knockdown in MCF7 cells cannot alter the expression of bone-related genes [132]. Additionally, the gene expression profile is different between MCF7-BM (derived from bone metastasis) and MDA-MB-231-BM cells established by intracaudal arterial injection, suggesting that the luminal breast cancer may have novel molecular mechanisms as compared to TNBC [67].

Due to a lack of HR+ models, little is known about the role of hormones in breast cancer metastasis or the hormone response to the organ-specific microenvironment [133]. However, increasing evidence has shown that the breast cancer subtype enables the prediction of an increased risk of site-specific metastasis. The study of metastasis in each subtype can provide a more comprehensive understanding and reference for clinical treatment [128,134]. The elucidation of molecular subtype-dependent mechanisms of metastasis is a major issue in future research, and it is necessary to construct a novel metastasis model for other subtypes.

## 5. Genomic Profiling of Breast Cancer

The analysis of genetic information from clinical samples has demonstrated the various metastatic patterns of breast cancer. Gene signatures that orchestrate the metastatic phenotype have been characterized. In 2000, Charlses M Perou et al. applied complementary DNA microarrays and reported 8102 human genes associated with estrogen receptor-negative and estrogen receptor-positive breast cancer according to their genetic patterns [135]. In 2002, Marc J van de Vijver et al. used an oligonucleotide microarray to characterize 295 young patients with or without breast cancer lymph node metastasis. The data revealed several gene signatures such as minichromosome maintenance complex component 6 (*MCM6*), *MMP9*, and cyclin E2 (*CCNE2*) that were associated with the risk of metastasis [44]. Transcriptome profiles also showed metastatic gene signatures associated with a poor prognosis in breast cancer patients. Based on the vast amount of work and the development of next generation sequencing, the cancer genome atlas (TCGA) sequenced and analyzed over 25 types of more than 10,000 primary tumors to characterize the DNA, RNA, and protein levels [136]. Moreover, the molecular taxonomy of breast cancer international consortium (METABRIC) cohort profiled the copy number, gene expression and proteome of 2000 primary tumors, and revealed the impact of copy number aberrations in breast cancer [137,138,139]. The primary tumor databases were frequently applied to the comparison with metastasis analysis. For example, Lucy R Yates et al. showed that the metastatic or disseminated tumor cells from breast cancer primary tumors acquired further mutation inactivation on switching defective (SWI)/sucrose nonfermenting (SNF) and JAK2-STAT3 pathways [140]. Moreover, Runpu Chen et al. applied static sample data from TCGA and METABRIC to study breast cancer progression. The mathematic model was able to demonstrate that the breast cancer subtypes can shift during metastasis [141].

However, it requires high bioinformatic technique to analyze the metastatic data from the primary tumor database. A more comprehensive analysis of metastatic cancers was performed by Dan R. Robinson et al. in 2017. They sequenced the genomic DNA and RNA of 500 metastatic specimens and released the MET500 dataset [142]. The MET500 dataset showed extensive genomic profiles of metastasis in clinical patients, and an epithelial-to-mesenchymal transition (EMT)-like inflammation signature and proliferation signature were observed in metastatic tissues.

Meanwhile, Yibin Kang et al. obtained the transcriptomic profile of MDA-MB-231 bone metastatic cells selected in vivo in 2003, and the osteolytic bone metastatic genes as mentioned in Section 3 were extracted [45], where they found that the genes of bone metastasis signatures were unidentified in previous breast cancer signatures of poor prognosis. This result raised the attention on the emergency of the profile of organ-tropic metastasis. Therefore, the lung metastatic in vivo selection was profiled by MDA-MB-231 and 1894 cells [25], as well as the brain metastasis in vivo selection by CN34 and MDA-MB-231 [91]. The organ-specific metastasis gene signatures were extracted based on these profiles. Brain and lung metastatic genes *COX2*, *EREG*, bone metastatic genes *CXCR4*, *MMP1*, etc., were identified and characterized. Yuxuan Han et al. established luminal-type bone metastatic cell line by MCF7 cells [67] and profiled luminal type breast cancer metastatic signatures such as trefoil factor 1 (*TFF1*), interferon alpha inducible protein 6 (*IFI6*) and galectin 1 (*LGALS1*), which were confirmed as metastasis-positive signatures [143] (Table 3). The high-throughput technologies have developed rapidly, and scientists are able to understand both the genomic and transcriptomic changes during cancer metastasis in clinical samples and experimental models.

The analysis of genetic information from clinical samples has demonstrated the various metastasis gene signature in breast cancer. Metastasis gene signatures that orchestrate the metastatic phenotype are characterized. From the establishment of novel highly metastatic cell lines and the identification of novel metastasis gene signatures, extensive clinical analysis with genomics and transcriptomics will be needed to identify their clinical significance. Most of the cohort studies have analyzed primary lesions, but it will be necessary to analyze the profile of metastatic lesions.

## 6. Conclusions

The in vivo selection method in breast cancer research has revealed many molecular mechanisms of metastasis and contributed to our understanding of cancer progression with distant metastasis. In this review, we discussed the characteristics of highly metastatic breast cancer models, including cancer cell lines and PDXs, with the view of methodology for establishment. Recent breast cancer metastasis studies suggest that even cancer cells that metastasize to the same organ show various metastatic cascades and patterns upon changing the transplantation method for in vivo selection. Also, various metastasis gene signatures can be revealed by changing the method and the molecular subtypes of breast cancer. However, most of the cancer cell line models have the limitation of the preclinical study. More establishment and magnification of the highly metastatic PDX models are necessary for preclinical metastasis study. On the other hand, some of the highly metastatic cancer cell line models have been also contributed to clinical research. Researchers will need to select an appropriate metastasis model by focusing not only on the metastatic organs but also on the metastatic cascade, mechanism and transplantation method. The appropriate selection of highly metastatic cancer cell line models and PDX models will be helpful for the elucidation of the breast cancer metastasis and the development of therapeutic strategies for metastasis.

## Figures and Tables

**Table 1:** List of highly metastatic PDX models in breast cancer.

PDX Models	Subtype	Method	Metastatic Organs	Reference
3887-LM	Basal-like (TNBC)	Orthotopic	Lung	[36]
HCI-002 LM2-1	TNBC	Orthotopic	Lung	[38]
Brain PDX (B0~B3)	TNBC	Intracranially	Brain	[39]
MFP PDX (M0~M3)	TNBC	Orthotopic	Brain	[39]

**Table 2:** List of highly metastatic breast cancer cell lines.

Cell Line	Species of Origin	Subtype	Method	Metastatic Organ	References
4T1-2776, 2792, 2869	Mouse	Basal-like (TNBC)	Spleen transplantation	Liver	[61]
4T1.3	Mouse	Basal-like (TNBC)	Orthotopic	Bone	[62]
4T1E/M3	Mouse	Basal-like (TNBC)	Intravenous	Bone	[63]
4T1LM	Mouse	Basal-like (TNBC)	Orthotopic	Liver	[64]
C3L5	Mouse	Unknown	Subcutaneous	Lung	[65]
CN34-LM1	Human	Claudin-low (TNBC)	Intravenous	Lung	[66]
MCF7-BM02	Human	Luminal A	Intracaudal arterial	Bone	[67]
MDA-MB-231-BM (BoM: 1833)	Human	Claudin-low (TNBC)	Intracardiac	Bone	[45]
MDA-MB-231-HM	Human	Claudin-low (TNBC)	Intravenous	Lung	[68]
MDA-MB-231-HM.LNm5	Human	Claudin-low (TNBC)	Orthotopic	Lymph node	[69]
MDA-MB-231-LM05	Human	Claudin-low (TNBC)	Orthotopic	Lung	[26]
MDA-MB-231-LM1-2-1	Human	Claudin-low (TNBC)	Intravenous	Lung	[26]
MDA-MB-231-LM2 (4175)	Human	Claudin-low (TNBC)	Intracardiac/Intravenous	Lung	[25]
MDA-MB-231-luc-D3H2LN	Human	Claudin-low (TNBC)	Orthotopic	Lymph node	[50]
MDA-MB-231HMLNm5	Human	Claudin-low (TNBC)	Orthotopic	Lung, Liver, Lymph node, Spleen and Paraspinal tissue	[69,70,71]
MDA-MB-468LN	Human	Basal-like (TNBC)	Orthotopic	Lymph node	[72]
mtMDA	Human	Claudin-low (TNBC)	Intracardiac	Bone	[73]
SUM159-M1a	Human	Claudin-low (TNBC)	Intravenous	Lung	[74]

Please see the Brain Metastasis Cell Lines Panel for highly metastatic brain cell (https://apps.cnio.es/app/BrainMetastasis/CellLines accessed on 11 January 2021) [58].

**Table 3:** Gene signatures of in vivo selection.

Metastasis Organ	Gene Signatures	Reference
Lung	EREG, CXCL1, MMP1, MMP2, SPARC, VCAM1, COX2	[25]
Lung	CD70, SOX4	[66]
Bone	IL1, CTGF, CXCR4, OPN	[45]
Bone	DKK1	[87]
Bone	S100A4	[73]
Brain	COX2, HBEGF, ST6GALNAC5	[91]
Brain	CTSS, L1CAM, GRIN2B, PCDH7, CX43	[18,95,96,97]
Liver	CLDN2	[61]

## Abbreviations

SCID	Severe combined immunodeficiency
NOD	Non-obese diabetic
GEMM	Genetically engineered mouse model
PDX	Patient derived xenograft
MetMap	Metastasis map
QOL	Quality of life
BBB	Blood-brain barrier
HR	Hormone receptor
HER2	Human epidermal growth factor receptor 2
ER	Estrogen receptor
PR	Progesterone receptor
TNBC	Triple negative breast cancer
EMT	Epithelial to mesenchymal transition
TCGA	The cancer genome atlas
METABRIC	Molecular taxonomy of breast cancer international consortium
